# *BHLH IRIDOID SYNTHESIS 3* is a member of a bHLH gene cluster regulating terpenoid indole alkaloid biosynthesis in *Catharanthus roseus*

**DOI:** 10.1002/pld3.305

**Published:** 2021-01-25

**Authors:** Sanjay Kumar Singh, Barunava Patra, Priyanka Paul, Yongliang Liu, Sitakanta Pattanaik, Ling Yuan

**Affiliations:** ^1^ Kentucky Tobacco Research & Development Center University of Kentucky Lexington KY USA; ^2^ Department of Plant and Soil Sciences University of Kentucky Lexington KY USA; ^3^ South China Botanical Garden Chinese Academy of Sciences Guangzhou China

**Keywords:** bHLH gene cluster, *Catharanthus roseus* (Madagascar periwinkle), terpenoid indole alkaloids, transcriptional regulation

## Abstract

Basic helix‐loop‐helix (bHLH) transcription factors (TFs) are key regulators of plant specialized metabolites, including terpenoid indole alkaloids (TIAs) in *Catharanthus roseus*. Two previously characterized subgroup‐IVa bHLH TFs, BIS1 (bHLH Iridoid Synthesis 1) and BIS2 regulate iridoid biosynthesis in the TIA pathway. We reanalyzed the recently updated *C. roseus* genome sequence and discovered that *BIS1* and *BIS2* are clustered on the same genomic scaffold with a previously uncharacterized bHLH gene, designated as *BIS3*. Only a few bHLH gene clusters have been studied to date. Comparative analysis of 49 genome sequences from different plant lineages revealed the presence of analogous bHLH clusters in core angiosperms, including the medicinal plants *Calotropis gigantea* (giant milkweed) and *Gelsemium sempervirens* (yellow jessamine), but not in the analyzed basal angiosperm and lower plants. Similar to the iridoid pathway genes, *BIS3* is highly expressed in roots and induced by methyl jasmonate. BIS3 activates the promoters of iridoid branch genes, *geraniol synthase* (*GES*), *geraniol 10‐hydroxylase* (*G10H*), *8‐hydroxygeraniol oxidoreductase* (*8HGO*), *iridoid synthase* (*IS*), *7‐deoxyloganetic acid glucosyl transferase* (*7‐DLGT*), and *7‐deoxyloganic acid hydroxylase* (*7DLH*), but not *iridoid oxidase* (*IO*). Transactivation of the promoters was abolished when BIS3 is converted to a dominant repressor by fusing with the ERF‐associated amphiphilic repression (EAR) sequence. In addition, BIS3 acts synergistically with BIS1 and BIS2 to activate the *G10H* promoter in tobacco cells. Mutation of the known bHLH TF binding motif, G‐box (CACGTG) in the *G10H* promoter significantly reduced but did not abolish the transactivation by BIS3. Promoter deletion analysis of *G10H* suggests that the sequences adjacent to the G‐box are also involved in the regulation by BIS3. Overexpression of *BIS3* in *C. roseus* flower petals significantly upregulated the expression of iridoid biosynthetic genes and increased loganic acid accumulation. *BIS2* expression was significantly induced by BIS3 although BIS3 did not directly activate the *BIS2* promoter. Our results advance our understanding of the regulation of plant specialized metabolites by bHLH TF clusters.

## 1 INTRODUCTION

Basic helix–loop–helix (bHLH) transcription factor (TF) genes are ancient regulatory genes present in all eukaryotic kingdoms and one of the largest TF families in plants (Feller et al., 2011). Plant bHLH TFs have been classified into 12 groups (I–XII), and each group is further subdivided into smaller orthologous subgroups (Heim et al., 2003). bHLH TFs have emerged as crucial components in the gene regulatory networks controlling many biological processes in plants, including light and phytohormone signaling (Kazan & Manners, 2013; Lee et al., 2006; Leivar et al., 2008; Yin et al., 2005), stress responses (Abe et al., 1997), shoot branching (Yang et al., 2012), and tissue and organ development (Kanaoka et al., 2008; Morohashi et al., 2007; Rajani & Sundaresan, 2001; Sorensen et al., 2003; Szécsi et al., 2006). In addition, bHLH TFs regulate the biosynthesis of plant specialized metabolites, such as nicotine in tobacco (Shoji & Hashimoto, 2011; Zhang et al., 2012), glucosinolates in Arabidopsis (Schweizer et al., 2013), cucurbitacin in cucumber (Shang et al., 2014), phytoalexins in rice (Yamamura et al., 2015), artemisinin in *Artemisia annua* (Shen et al., 2016), paclitaxel in *Taxus cuspidata* (Lenka et al., 2015), saponins in *Medicago truncatula* (Mertens, Pollier, et al., 2016; Ribeiro et al., 2020), amygdalin in almond (Sanchez‐Perez et al., 2019), and anthocyanins in many plant species (Patra et al., 2013; Xu et al., 2015). The bHLH TFs belonging to subgroup IIId, IIIe, and IIIf are well characterized for their roles in plant specialized metabolite biosynthesis. For instance, the subgroup‐IIIe (e.g., MYC2‐type) and ‐IIIf (e.g., GL3, EGL3, and TT8) bHLH TFs are positive regulators (Patra et al., 2013), whereas those of subgroup‐IIId (bHLH03, bHLH13, bHLH14, and bHLH17) are negative regulators, of flavonoid biosynthesis in *Arabidopsis* (Sasaki‐Sekimoto et al., 2013; Song et al., 2013). Two subgroup‐Ib bHLH, Bl (bitter leaf) and Bt (bitter fruit) regulate biosynthesis of cucurbitacin in cucumber (Shang et al., 2014). The group‐IV bHLH TFs are divided into four relatively less studied subgroups (IVa‐d). In *Arabidopsis*, members of subgroup‐IVa (bHLH18, bHLH19, bHLH20, and bHLH25) regulate iron homeostasis (Cui et al., 2018). A few subgroup‐IVa bHLH TFs are key regulators of terpene biosynthesis in plants. Triterpene Saponin Biosynthesis Activating Regulator 1 (TSAR1), TSAR2, and TSAR3 regulate the biosynthesis of the triterpene saponins in *M. truncatula* (Mertens, Pollier, et al., 2016; Ribeiro et al., 2020). In *Chenopodium quinoa* (quinoa), two TSAR‐like TFs, TSARL1 and TSARL2, regulate saponin biosynthesis (Jarvis et al., 2017). Information is limited on the subgroup‐IV bHLH TFs for the regulation of biosynthesis of specialized metabolites.

*Cathuranthus roseus* is the exclusive source of almost 200 terpenoid indole alkaloids (TIAs), including the therapeutic compounds vincristine and vinblastine (De Luca et al., 2014). TIA biosynthesis involves more than 30 different enzymes and takes place in at least four different cell types and subcellular compartments (Courdavault et al., 2014; Patra et al., 2013). TIAs are derived from two distinct pathways: the shikimate pathway generates the indole moiety, tryptamine, and the methylerythritol phosphate (MEP)‐derived iridoid pathway provides the terpenoid moiety secologanin. Condensation of tryptamine and secologanin yields strictosidine that serves as a precursor for numerous more complex TIAs (Thamm et al., 2016). Studies by independent research laboratories over the years have identified a number of TFs that regulate TIA biosynthesis in response to phytohormones and other abiotic factors. These TFs include transcriptional activators from the TF families of AP2/ERFs (ORCA2/3/4/5/6) (van der Fits & Memelink, 2000; Li et al., 2013; Menke et al., 1999; Paul et al., 2017, 2020; Singh et al., 2020), bHLH (CrMYC2, BIS1 and 2) (Schweizer et al., 2018; Van Moerkercke et al., 2015, 2016; Zhang et al., 2011), WRKY (CrWRKY1) (Suttipanta et al., 2011), and GATA (CrGATA1) (Liu et al., 2019), as well as transcriptional repressors from the families of basic leucine zipper (bZIP) GBF1/2 (Sui et al., 2018), bHLH (RMT1 and PIF1) (Liu et al., 2019; Patra et al., 2018) and zinc finger factors (ZCT1/2/3) (Pauw et al., 2004). However, compared to the discovery of almost the complete set of genes encoding TIA pathway enzymes, fewer regulatory proteins have been identified and characterized.

Genomic sequence analysis has revealed that a subset of TFs involved in specialized metabolite biosynthesis is present as clusters in plant genomes. In *C. roseus*, five AP2/ERFs, ORCA2, ORCA3, ORCA4, ORCA5, and ORCA6, reside on the same genomic scaffold to form a cluster (Kellner et al., 2015; Paul et al., 2017; Singh et al., 2020). Analogous AP2/ERF gene clusters have also been identified and characterized in tobacco for nicotine biosynthesis (Kajikawa et al., 2017; Shoji et al., 2010) and in tomato (Cardenas et al., 2016; Nakayasu et al., 2018; Thagun et al., 2016) and potato (Cardenas et al., 2016) for the biosynthesis of steroidal glycoalkaloids (SGAs). In petunia, an R2R3 MYB TF cluster controls anthocyanin biosynthesis (Zhang et al., 2019). A member of an almond bHLH TF cluster (bHLH1 to bHLH5) is involved in the biosynthesis of amygdalin (Sanchez‐Perez et al., 2019). Similarly, two bHLH factors, Bl and Bt, involved in cucurbitacin biosynthesis, are part of a small bHLH TF cluster (Shang et al., 2014). TF clusters comprise homologous TF genes in tandem orders with overlapping or unique functions. As demonstrated in ERF clusters in *C. roseus* and tobacco, a conserved mechanism allows the individual TFs within a cluster to regulate each other (Paul et al., 2020), with some individuals playing more dominant roles in regulating the pathway than others in the same cluster (Shoji & Yuan, 2021; Yuan, 2020). Our current understanding of the origin, numbers, and evolution of TF clusters remains scant. We, therefore, endeavored to explore the recently updated *C. roseus* genome sequence to identify additional candidate TF clusters involved in TIA biosynthesis.

Two bHLH TFs, BIS1, and BIS2, regulate the iridoid branch of the TIA pathway (Van Moerkercke et al., 2015, 2016). We discovered that *BIS1* and *BIS2* are present in the same genomic scaffold in the *C. roseus* genome along with a previously uncharacterized bHLH TF, designated as *BIS3*. Genomic sequence analyses revealed the presence of analogous bHLH clusters in a wide range of plant species, including Arabidopsis and several medicinal plants. Transcriptomic analysis revealed that spatial expression of clustered *BIS* genes and iridoid pathway genes is highly correlated and root‐specific. Similar to *BIS1* and *BIS2*, *BIS3* expression is induced by methyl jasmonate (MeJA). Transient overexpression of *BIS3* in *C. roseus* flower petals significantly upregulated the iridoid pathway genes. In addition, BIS3 activates the key iridoid pathway gene promoters in plant cells. Our findings provide new insights into bHLH TF clusters involved in the biosynthesis of plant specialized metabolites.

## 2 MATERIALS AND METHODS

### 2.1 Plant materials

*Catharanthus roseus* (L.) G. Don var. “Little Bright Eye” (NE Seed) was used for gene expression, cloning, and transient overexpression in flower petals. *Nicotiana tabacum* var. Xanthi cell line was used for protoplast‐based transient assays. Tobacco cell suspension cultures were maintained at 26°C in dark by gentle shaking (120 rpm). *C. roseus* plants were maintained in the growth room under 16/8 hr photoperiod at 24°C.

### 2.2 Construction of plant expression vector and *Agrobacterium* infiltration of *C. roseus* petals

For transient overexpression in flower petals, *BIS3* was amplified using PCR from *C. roseus* seedling cDNA and cloned into the pCAMBIA2301 vector containing the *CaMV*35S promoter and the *rbcS* terminator (Pattanaik, Kong, et al., 2010). Construction pCAMBIA2301‐*ORCA5* was described previously (Paul et al., 2020). The pCAMBIA2301 vector alone was used as an empty vector (EV) control. The plasmids were transformed into *Agrobacterium tumefaciens GV3101* by the freeze‐thaw method. All open flowers in the plants were removed, and flower buds opened the next day were used for transformation. *Agrobacterium* infiltration of *C. roseus* flower petals was performed as previously described (Schweizer et al., 2018). Flower petals uniformly infiltrated with *Agrobacterium* were collected after 48h for the measurement of gene expression and metabolites. Data presented here are from three independent biological replicates.

### 2.3 RNA isolation and cDNA synthesis

*C. roseus* seeds were surface‐sterilized as described previously (Patra et al., 2018) and then germinated on half‐strength solid Murashige and Skoog (MS) medium (Caisson Labs). Two‐week‐old axenic seedlings were immersed in half‐strength MS medium with 100 µM methyl jasmonate (MeJA) for 2 hr. Mock‐treated seedlings were used as control. Total RNA was isolated from the seedlings, digested with DNase, and used for first‐strand cDNA synthesis as described previously (Paul et al., 2017).

### 2.4 Tobacco protoplast isolation and electroporation

The reporter plasmids for transient protoplast assays were generated by cloning the *GES*, *G10H*, *8HGO*, *IS*, *IO*, *7‐DLGT*, and *7DLH* promoters upstream of a firefly *luciferase* (*LUC*) reporter and *rbcS* terminator. The G‐box motif (CACGTG) in the *G10H* promoter was mutated using site‐directed mutagenesis as previously described (Pattanaik et al., 2010). The generation of deletion fragments of *G10H* promoter using PCR was described previously (Suttipanta et al., 2007). The *GUS* reporter in *G10H* promoter fragments was replaced by *LUC*. The effector plasmids were made by cloning *BIS1*, *BIS2*, and *BIS3* into a modified pBS vector under the control of the *CaMV*35S promoter and *rbcS* terminator. The 12 amino acid (LDLDLELRLGFA) EAR (ERF‐associated amphiphilic repression) motif (also know as SRDX) (Hiratsu et al., 2003) was fused to the 3'‐end of *BIS3* to generate BIS3‐SRDX. The ß‐glucuronidase (*GUS*) reporter driven by the *CaMV*35S promoter and *rbcS* terminator was used as an internal control. Protoplast isolation from tobacco cell suspension cultures and electroporation with plasmid DNA were performed as previously described. The reporter (promoter‐*LUC*) plasmid alone or in combination with the effectors were electroporated into tobacco protoplasts as previously described (Pattanaik, Werkman, et al., 2010). An aliquote of 750 µl containing approximately 2 × 10^6^ protoplasts were used for each electroporation. Luciferase and GUS activities in transfected protoplasts were measured as described previously (Pattanaik, Werkman, et al., 2010). Each experiment was repeated three times.

### 2.5 Real‐time quantitative PCR

Real‐time quantitative PCR (RT‐qPCR) was performed as described previously (Suttipanta et al., 2011). All PCRs were performed in triplicate and repeated at least three times. Total RNA isolated from *C. roseus* seedlings, or flower petals infiltrated with the empty‐vector, *BIS3*, or *ORCA5* was digested with DNase and used for cDNA synthesis and RT‐qPCR as previously described (Patra et al., 2018; Paul et al., 2017). The comparative cycle threshold (Ct) method was used to measure transcript levels. In addition to the *C. roseus Elongation Factor 1∞* (*EF1∞*), 40S *Ribosomal Protein S9* (*RPS9*) gene, was used as a second internal control (Liscombe et al., 2010). The primers are listed in Table S1.

### 2.6 Bioinformatic analysis

Transcriptomic data from five different tissues (flower, immature leaf, mature leaf, stem, and root) were obtained from the NCBI sequence read archive database (accession number SRA030483). Raw reads were processed and reads per kilobase of transcript per million mapped reads (RPKM) value was calculated as described previously (Singh et al., 2015). The gene expression correlation matrices for the *BISs* were calculated and visualized with the corrplot R package (Wei & Simko, 2017). Heatmap analyses of *BISs* expression in different tissues were carried out using the pheatmap package with euclidean distance and complete linkage as distance measure and clustering methods (Kolde & Kolde, 2015).

The Neighbor‐Joining (NJ) tree was constructed based on a MAFFT v7 alignment and visualized by Evolview v3 (Subramanian et al., 2019). Gene location was visualized using the Gview (Petkau et al., 2010). PLAZA database, a versatile resource to study comparative genomics and to analyze the evolution of gene families in the green plant lineages (Van Bel et al., 2018), was used to get the information on the genomic organization of BIS homologs in other plant species.

### 2.7 Alkaloid analysis

Alkaloid extracted from flower petals infiltrated with empty vector or *BIS3* were analyzed as previously described (Patra et al., 2018; Singh et al., 2020).

### 2.8 Statistical analysis

The data presented here were statistically analyzed by Student's *t* test or one‐way analysis of variance (ANOVA), and Tukey's Honestly Significant Difference (HSD) for multiple comparisons.

## 3 RESULTS

### 3.1 Genome sequence analysis revealed the *BIS* TF cluster in *C. roseus* genome

**FIGURE 1:**
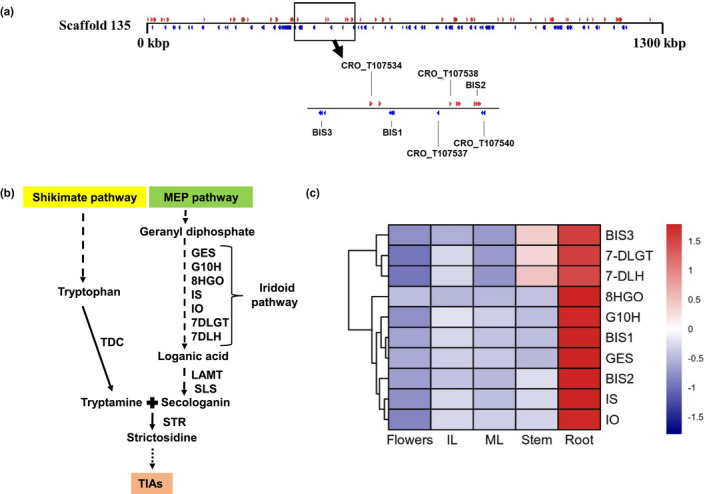
Characterization of the BISs gene cluster. (a) Organization of the BIS gene clusters in *C. roseus* genome. Genes and their orientations are depicted using RED and BLUE color arrows: RED, genes on the sense strand; BLUE, genes on the antisense strand. (b) A simplified diagram of TIA pathway in *C. roseus*: 7DLGT, 7‐deoxyloganetic acid glucosyl transferase; 7DLH, 7‐deoxyloganic acid hydroxylase; 8HGO, 8‐hydroxygeraniol oxidoreductase; G10H, geraniol 10‐hydroxylase; GES, geraniol synthase; LAMT, loganic acid *O*‐methyl transferase; IO, iridoid oxidase; IS, iridoid synthase; SLS, secologanin synthase; STR, strictosidine synthase; TDC, tryptophan decarboxylase (c) Expression of *BISs* and iridoid pathway genes in various tissues. Heatmap showing the normalized RNA‐seq (Accession no. SRA030483) expression values in reads per kilobase of transcript per million mapped reads (RPKM) transformed into Z‐score. Hierarchical clustering was conducted in R using the pheatmap package based on Euclidean distance with complete linkage rule. The color key at the side represents row‐wise Z‐score

Previously, two subgroup‐IVa bHLH TFs, BIS1, and BIS2, have been reported as the regulators of iridoid branch of the TIA pathway in *C. roseus* (Van Moerkercke et al., 2015, 2016). The two known *BIS* genes were used as queries in our analysis to reveal the putative location of a *bHLH* gene cluster on scaffold 135. Further analysis showed that the >1,300‐kb long scaffold 135 contains three *bHLH* genes in a <100‐kb region of the scaffold. In addition to *BIS1* and *BIS2*, an uncharacterized *bHLH* gene, designated here as *BIS3* (GenBank accession no. MN646782), was found in close proximity to the same scaffold (Figure 1a).

### 3.2 Subgroup‐IVa bHLH TF cluster is common in core angiosperms

**FIGURE 2:**
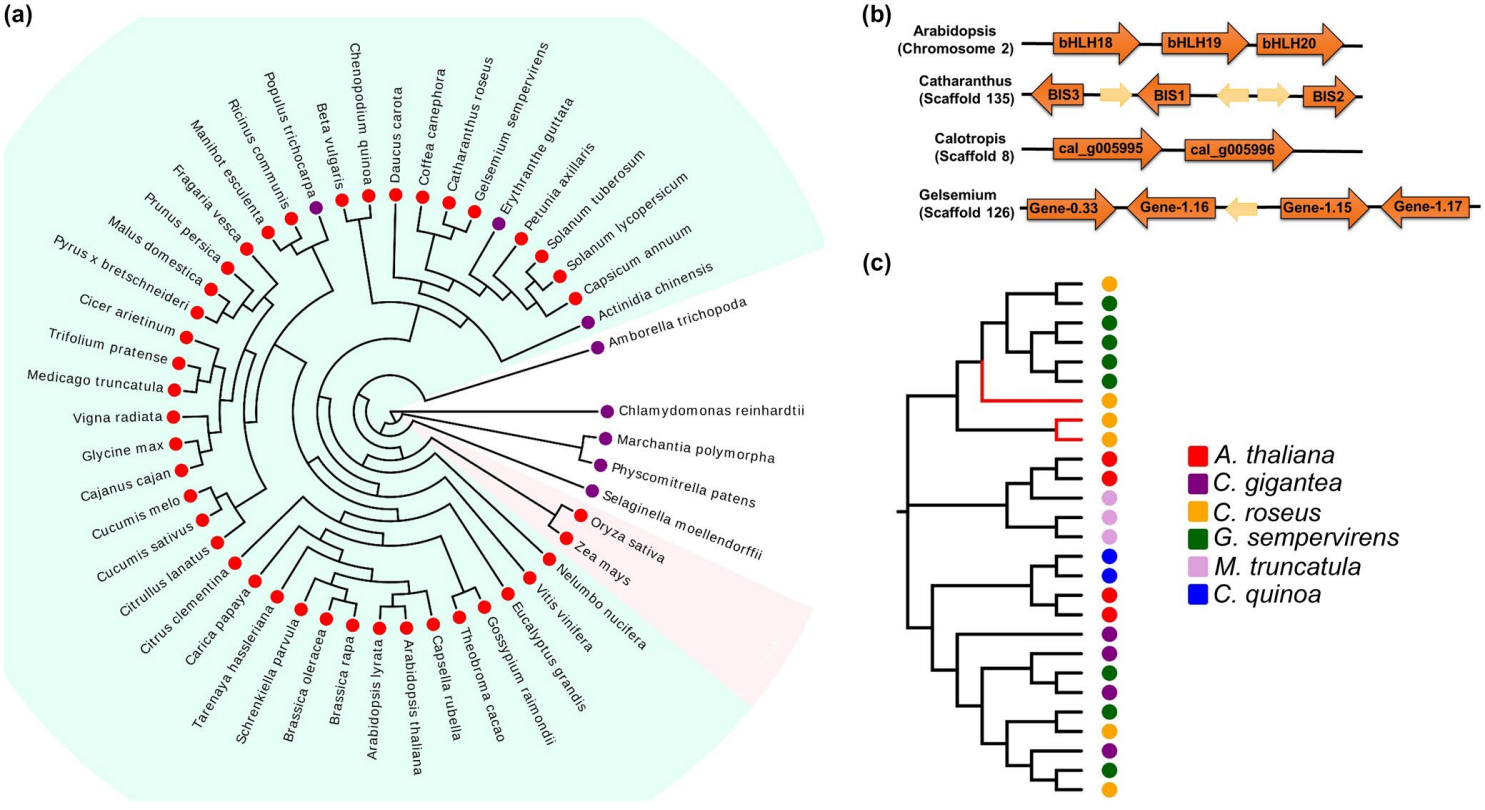
The clade IV bHLHs in plants (a) Species tree showing the distribution of bHLH subgroup IVa gene cluster in different plants. The presence and absence of gene clusters are indicated by *red* and *purple* circles, respectively. The clade with monocot plants is highlighted with a light *pink* background, while dicot‐specific clades are with *aquamarine* background. (b) A schematic representation of the subgroup IVa bHLH clusters in *Catharanthus roseus* and their orthologs in *Calotropis gigantea*, *Gelsemium sempervirens,* and *Arabidopsis thaliana*. (c) Phylogenetic analysis of bHLH subgroup IVa transcription factors from *C. roseus*, *C. gigantea*, *G. sempervirens*, *A. thaliana*, *Medicago truncatula* and *Chenopodium quinoa*. The BISs‐specific clade is highlighted in red color

To determine the origin, distribution, and species‐specific expansions or reductions of the subgroup‐IVa bHLH TF cluster during the course of green plant evolution, we performed a comparative genomic analysis of 49 green plants from different lineages that include 42 eudicots, two monocots (rice and maize), the basal angiosperm (*Amborella trichopoda*), one lycophyte (*Selaginella moellendorffii*), liverwort (*Marchantia polymorpha*), moss (*Physcomitrella patens*), and one chlorophyte (*Chlamydomonas reinhardtii*) (Figure 2a). We did not find such bHLH gene cluster in lower plant lineages, such as *C. reinhardtii*, *M. polymorpha*, *P. patens*, *S. moellendorffii*, and *A. trichopoda*. Subgroup‐IVa bHLH TF clusters were found in most of the analyzed monocot and dicot genomes, with a few exceptions, such as *Erythranthe guttata* (Phrymaceae) and *Populus trichocarpa* (Salicaceae). The numbers of genes in the subgroup‐IVa bHLH clusters vary greatly from two (*Chenopodium quinoa*, *Calotropis gigantea)*, three (Arabidopsis and *C. roseus*) to seven (*Trifolium pratense*, *Eucalyptus grandis*). The *bHLH* genes in a cluster are either arranged in tandem (as in *Arabidopsis*) or interrupted by one or more genes (as in *C. roseus*) (Figure 2b). These findings indicate that subgroup‐IVa bHLH clusters possibly have appeared after the separation of the mesangiosperms (core angiosperms) from the basal angiosperm, *A. trichopoda,* and other lower plants, and further expansion or reduction occurred at the genus level in the core angiosperm lineage.

### 3.3 Phylogenetic analysis of subgroup‐IVa bHLH cluster

In *Arabidopsis*, three (bHLH18, bHLH19, bHLH20) of the four subgroup‐IVa bHLH TFs form a cluster that regulates the iron deficiency responses and homeostasis by interacting with other bHLH TFs (Cui et al., 2018). In *C. roseus*, the subgroup‐IVa contains six bHLH TFs, and only *BIS1*, *BIS2*, and *BIS3* are clustered in the same genomic scaffold, while the other three are present in different scaffolds. We also found subgroup‐IVa bHLH gene clusters in the genomes of two recently sequenced medicinal plants, *C. gigantea* (Apocynaceae) and *Gelsemium sempervirens* (Gelsemiaceae). *C. gigantea* is the source of the anticancer and antimalarial cardenolides (Hoopes et al., 2018) and found to contain a cluster of two bHLH genes. *G. sempervirens* is related to *C. roseus* and produces oxyindole alkaloids (Franke et al., 2019). *G. sempervirens* genome harbors a cluster of three bHLH genes that are homologous to BISs (Figure 2b). Unlike the *C. roseus* and *G. sempervirens* bHLH clusters, which are interrupted by other non‐homologous genes, the two bHLH genes in *C. gigantea* are arranged in a tandem array (Figure 2b). In *C. roseus*, one gene (*CRO_T107534*) is located in between *BIS1* and *BIS3* and two genes (*CRO_T107537*, *CRO_T107538*) are present in between *BIS1* and *BIS2*. *CRO_T107534*, *CRO_T107537*, and *CRO_T107538* are annotated as hypothetical proteins in *C. roseus* genome. Next, we performed phylogenetic analysis of the subgroup‐IVa bHLH TFs from *Arabidopsis*, *C. roseus*, *M. truncatula*, *C. quinoa*, *G. sempervirens*, and *C. gigantean*. The three members of the BIS cluster are grouped together in a sub‐clade, whereas three other non‐clustered *C. roseus* IVa bHLH TFs are present in different sub‐clades (Figure 2c).

### 3.4 BIS TFs share limited sequence identity but a similar expression profile

Amino acid sequence analysis showed that all three BIS contain a highly conserved bHLH DNA binding domain (Figure S1). BIS3 shares approximately 39% amino acid sequence identity with BIS1 and 85% identity with BIS2. Analysis of the genomic sequence revealed that both *BIS1* and *BIS2* harbor four exons and three introns whereas the *BIS3* gene harbors five exons interrupted by four introns (Figure S1). Next, we analyzed the expression profiles of the *BIS* cluster genes and the iridoid pathway genes (Figure 1b). We used the publicly available transcriptomic data from different tissues (immature leaf, mature leaf, stem, root and flower; accession number SRA030483) to examine gene expression. Similar to *BIS1* and *BIS2*, expression of *BIS3* was significantly higher in the roots compared to other tissues (Figure 1c). Co‐expression analysis showed that, similar to all *BISs*, iridoid pathway genes are highly expressed in roots (Figure 1c).

### 3.5 *BIS3* expression is induced by methyl jasmonate

**FIGURE 3:**
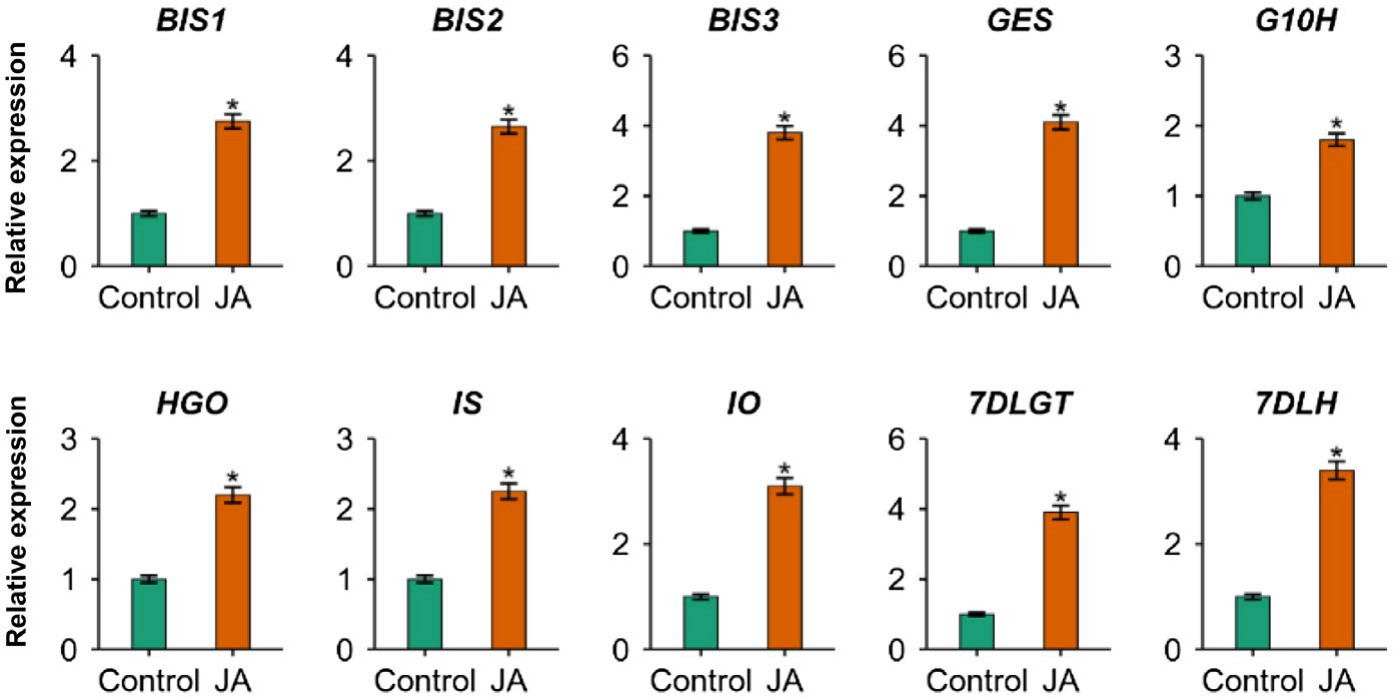
Expression of *BISs* and iridoid pathway genes in response to MeJA. Ten‐day‐old *C. roseus* seedlings were treated with 100 μM MeJA (JA) for 2 hr and gene expression in the whole seedling was measured by RT‐qPCR. Mock‐treated seedlings were used as controls. Data represent means ± SDs of three biological samples. Statistical significance was calculated using the Student's *t* test: *p < .05

MeJA is a major elicitor of a wide range of plant specialized metabolites, including anthocyanins, nicotine, TIA, glucosinolates (GS), benzophenanthridine alkaloids, flavonoids, and artemisinin (Wasternack & Strnad, 2019). Members of the subgroup‐IVa bHLH TF families are induced by MeJA in *M. truncatula* and *C. roseus* (Mertens et al., 2016). To determine the temporal expression patterns of the *BIS3* in response to MeJA, we treated *C. roseus* seedlings with 100 µM MeJA for 2 hr and measured gene expression using RT‐qPCR. As shown in Figure 3, the expression of all three *BISs* was significantly induced (~3‐ to 4‐fold) by MeJA. The sequential conversion of geranyl diphosphate to loganic acid is catalyzed by seven enzymes. The transcript levels of genes encoding these seven enzymes were also induced by 2‐ to 4‐fold after 2 hr of MeJA treatment (Figure 3). Collectively, these findings suggest that MeJA positively affects *BIS3* expression along with other iridoid pathway genes and regulators.

### 3.6 BIS3 transactivates the iridoid pathway gene promoters in tobacco cells

**FIGURE 4:**
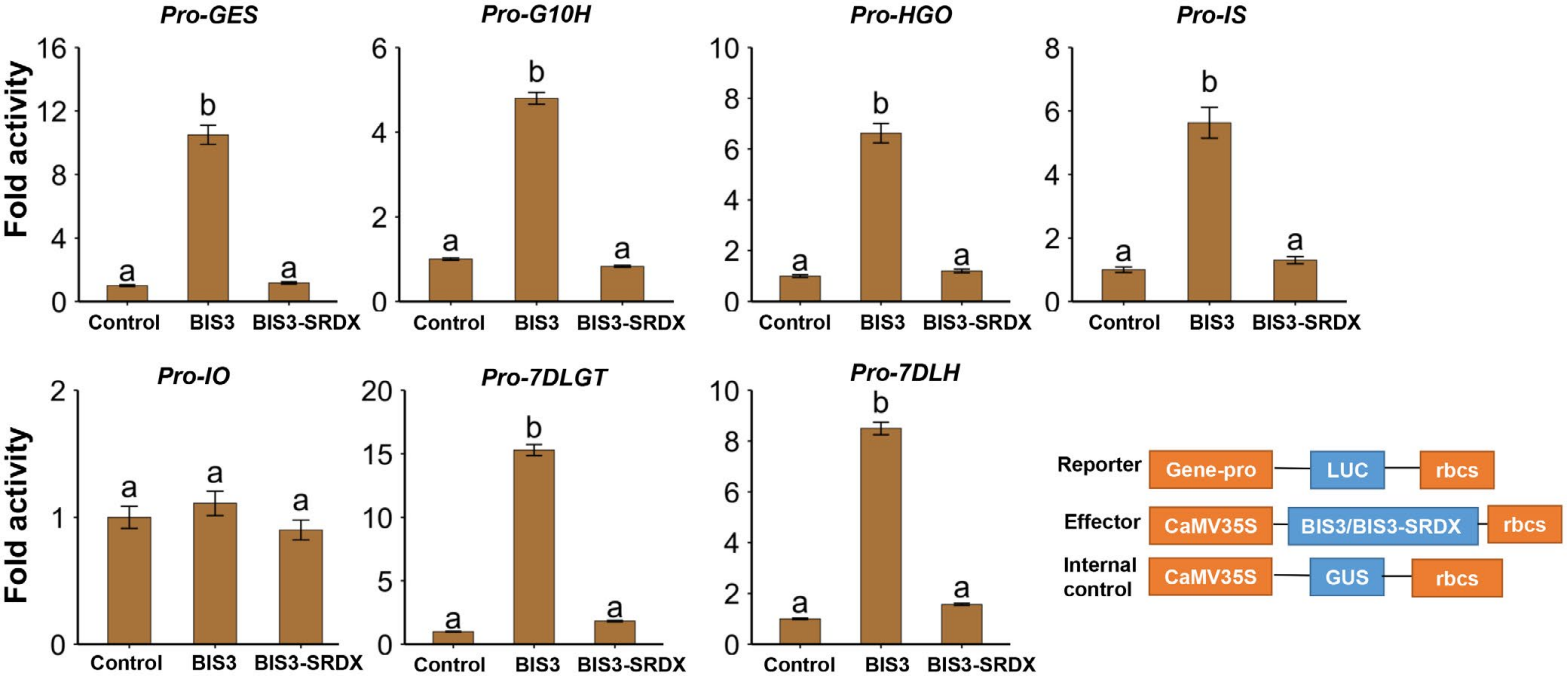
Transactivation of iridoid pathway promoters by BIS3 in tobacco cells. Schematic diagrams of plasmids used in transactivation assay. The *GES*, *G10H*, *8HGO*, *IS*, *IO*, *7DLGT*, and *7DLH* promoters fused to *luciferase* (*LUC*) reporter were electroporated into tobacco protoplasts either alone or with an effector plasmid (*BIS3*). The *CaMV*35S‐*GUS* reporter was used as an internal control. Luciferase activity was normalized against GUS activity. Control represents the reporter alone without effectors. Data represent means ± *SD*s of three biological samples. Different letters denote statistical differences as assessed by one‐way ANOVA and Tukey HSD test, *p* < .05. Schematic diagrams of the plasmid constructs used in transactivation assay (bottom panel)

To determine whether BIS3 can activate the iridoid biosynthetic genes, we performed promoter transactivation assays using BIS3 in tobacco protoplasts (Figure 4). The promoters of *GES*, *G10H*, *HGO*, *IS*, *IO*, *7DLGT* or *7DLH* fused to firefly *luciferase* (*LUC*) reporter gene was electroporated into tobacco protoplasts alone or in combination with *BIS3*. Luciferase activities were significantly higher (4.8‐ to 15‐fold) when BIS3 was coexpressed with *GES*, *G10H*, *HGO*, *IS 7DLGT* or *7DLH* promoter‐reporter plasmid (Figure 4). We did not detect a significant change in the transactivation of the *IO* promoter by BIS3 (Figure 4). To further elucidate the function of BIS3 in the activation of iridoid pathway genes, we converted BIS3 to a dominant repressor (BIS3‐SRDX) by fusing it with the 12 amino acid (LDLDLELRLGFA) EAR (ERF‐associated amphiphilic repression) motif (also know as SRDX) (Hiratsu et al., 2003). As shown in Figure 4, BIS3‐SRDX was unable to transactivate the *GES*, *G10H*, *8HGO*, *IS*, *7DLGT* or *7DLH* promoters in the protoplast assay. The basal activity of the promoters is presumably due to basal transcription factors present in tobacco cells. To determine whether BIS3 acts synergistically with BIS1 or BIS2 to regulate the iridoid pathway genes, we compared the transactivation activity of the *G10H* promoter by BIS1, BIS2 or BIS3 alone or in combination in tobacco cells. As expected, BIS1 and BIS2 significantly activated the *G10H* promoter in tobacco cells; however, the transactivation activity was increased significantly when BIS3 was co‐electroporated with BIS1 or BIS2 (Figure S2), suggesting that BIS cluster TFs act together to regulate the *G10H* promoter.

### 3.7 The G‐box motif and adjacent sequences in the *G10H* promoter are critical for transactivation by BIS3

**FIGURE 5:**
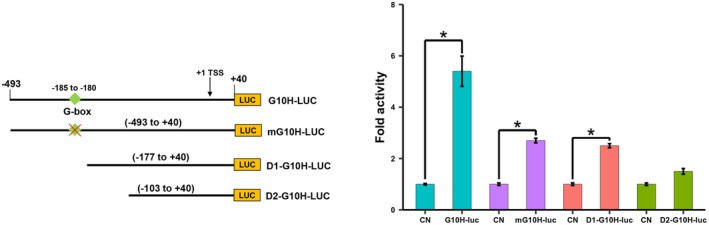
Transactivation of the *G10H* promoter mutant and deletion fragment by BIS3 in tobacco cells. Schematic diagrams of the *G10H* promoter fragments used in transactivation assay (left panel). The mutant or deleted fragments of *G10H* promoters fused to *luciferase* (*LUC*) reporter were electroporated into tobacco protoplasts either alone or with an effector plasmid (*BIS3*). *CaMV*35S‐*GUS* reporter served as an internal control. Luciferase activity was normalized against GUS activity. Control represents the reporter alone without effectors. Data represent means ± SDs of three biological samples. Statistical significance was calculated using the Student's *t* test: *p < .05; CN, control (reporter only)

The conversion geraniol to 10‐hydroxy geraniol, catalyzed by G10H, is the first committed step in iridoid biosynthesis in *C. roseus* (Thamm et al., 2016). We have previously isolated and characterized the *G10H* promoter in *C. roseus* hairy roots and transgenic tobacco plants (Suttipanta et al., 2007). The 533 bp *G10H* promoter (−497 to +40 relative to the transcription start site; TSS) used in our study contains only one canonical G‐box motif (CACGTG) at position −185 to −180 relative to the transcription start site (TSS). bHLH TFs are known to bind E‐box (CANNTG), G‐box or variants in the target promoters (Patra et al., 2018). Recent studies have shown that the subgroup‐IV bHLH TFs also bind a variant of G‐box motif, N‐box (CACGAG), in the promoters (Mertens, Pollier, et al., 2016; Yamamura et al., 2015). Although the *G10H* promoter does not contain an N‐box motif (Suttipanta et al., 2007), it is significantly activated by BIS1 (Figure S2) (Van Moerkercke et al., 2015), BIS2 (Van Moerkercke et al., 2016) and BIS3 (this study; Figure 4 and Figure S2). We, therefore, mutated the G‐box (CACGTG) to CAAAAA and evaluated the transactivation of the mutant promoter by BIS3 in tobacco cells. Mutation of G‐box motif reduced but did not abolish the transactivation of the *G10H* promoter by BIS3. To further determine the sequence motifs involved in BIS3 regulation, we generated two deletion fragments of the *G10H* promoter (−177 to +40 and −103 to +40 relative to TSS) that do not contain known bHLH binding motifs and measured their transactivation activities by BIS3 in tobacco cells. Similar to mutation, deletion of the G‐box motif in D1‐G10H (−177 to +40) did not abolished transactivation by BIS3 (Figure 5). However, further deletion D2‐G10H (−103 to +40) of the *G10H* promoter almost abolished the transactivation, suggesting that additional sequences (~70 bps adjacent to the G‐box) in the promoter potentially contribute to the transactivation of *G10H* by BIS3.

### 3.8 *BIS3* overexpression in *C. roseus* flower petal upregulates TIA pathway gene expression

**FIGURE 6:**
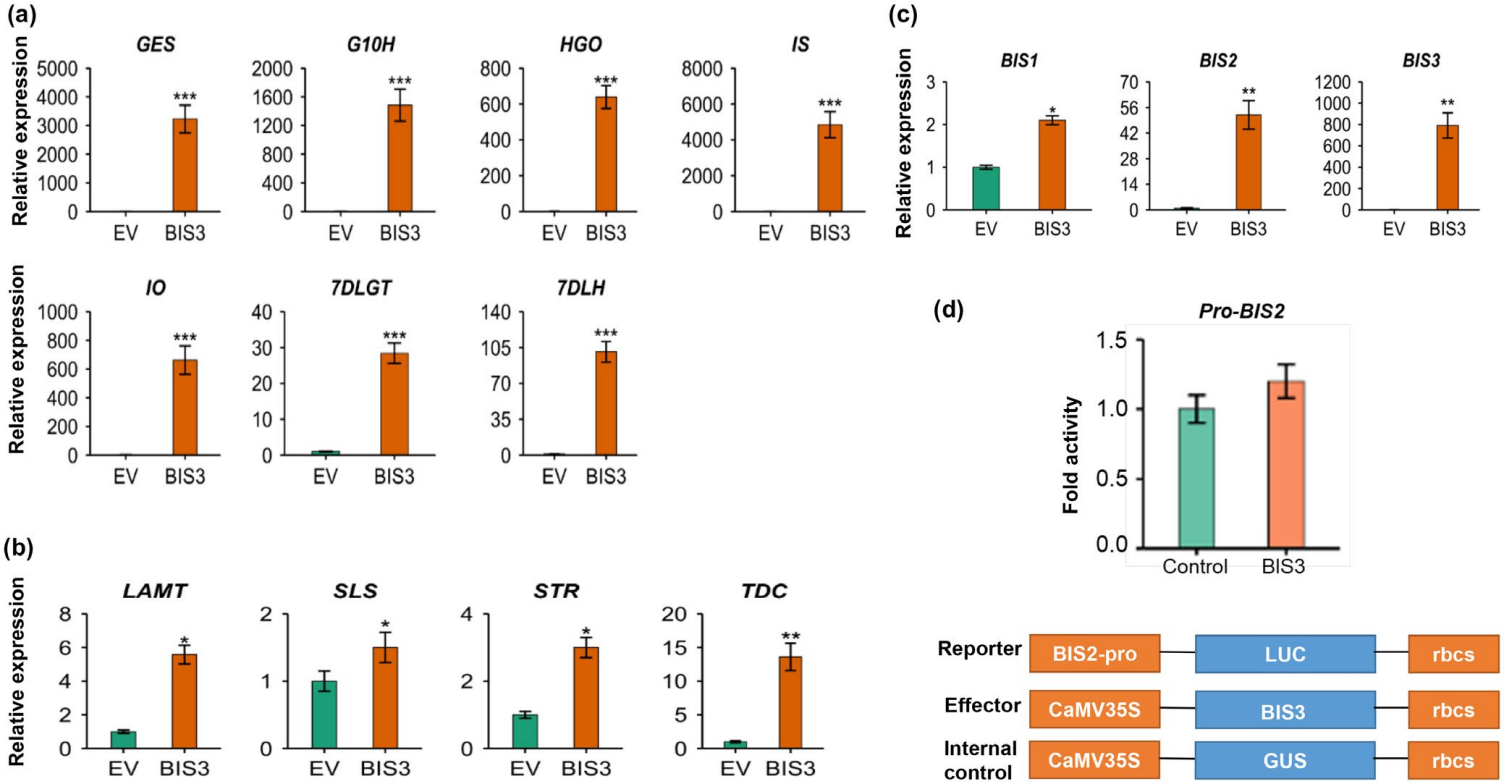
Expression of TIA pathway genes in *BIS3*‐overexpressing flower petals. Expression of iridoid (a), other TIA pathway (b) and *BIS1*, *BIS2,* and *BIS3* (c) in *BIS3*‐overexpressing flower petals as measured by RT‐qPCR. (d) Transactivation of *BIS2* promoter by BIS3 in tobacco cells. *BIS2* promoter fused to *luciferase* (*LUC*) reporter were electroporated into tobacco protoplasts either alone or with *BIS3*. *CaMV*35S‐*GUS* reporter served as an internal control. Luciferase activity was normalized against GUS activity. Control represents the reporter alone without effectors.The error bars represent the means ± *SD* from three biological replicates. Statistical significance was calculated using the Student's *t* test: *p < .05; **p < .01, ***p < .001

To further substantiate the role of BIS3 in the regulation of the TIA pathway, we transiently overexpressed *BIS3* in *C. roseus* flower petals as previously described (Schweizer et al., 2018; Singh et al., 2020) and measured the expression of TIA pathway genes by RT‐qPCR. As shown in Figure 6a, expression of all iridoid pathway genes were significantly upregulated (25‐ to 4,500‐fold) compared to the empty vector (EV) control. Induced expression of *IS* was the highest, while that of *7DLGT* was the lowest. Although BIS3 does not activate the *IO* promoter in the tobacco protoplast assay (Figure 4), *IO* expression was increased by 600‐fold compared to EV in flower petals overexpressing *BIS3* (Figure 6a). The conversion of loganic acid to secologanin is catalyzed by loganic acid *O*‐methyltransferase (LAMT) and secologanin synthase (SLS), which are regulated the ORCAs (Paul et al., 2020; Van Moerkercke et al., 2015). *BIS3* overexpression moderately upregulated *LAMT* (5‐fold) and *SLS* (1.5‐fold) expression. In addition, expression of *TDC* (*tryptophan decarboxylase*) and *STR* (*strictosidine synthase*), which are direct targets of ORCAs, was increased in *BIS3*‐overexpressing petals compared to EV (Figure 6b). To determine whether BIS3 regulates or is regulated by ORCAs, we measured the expression of three *BIS* in *ORCA5*‐overexpressing flower petals. Expression of *BIS1* and *BIS2* was induced by 4‐ and 15‐fold, respectively, whereas *BIS3* expression remained unchanged (Figure S3a). In addition, we measured the expression of all five *ORCAs* in *BIS3*‐overexpressing flower petals. Expression of *ORCA2* showed modest induction (~ 6‐fold), whereas that of *ORCA3* and *ORCA5* reduced significantly; *ORCA4* and *ORCA6* expression was not significantly altered by BIS3 (Figure S3b).

Previous studies have shown that *BIS2* expression was induced in *BIS1*‐overexpressing hairy roots and flower petals (Schweizer et al., 2018; Van Moerkercke et al., 2016). To determine whether *BIS3* overexpression activates similar amplification loops, we measured the expression of *BIS1* and *BIS2* in *BIS3*‐overexpressing flower petals. *BIS2* expression was increased by more than 50‐fold, whereas that of *BIS1* was induced by 2‐fold in *BIS3*‐overexpressing flower petals (Figure 6c). To further determine whether BIS3 can directly activate *BIS2* expression, we cloned the *BIS2* promoter and fused it to the *LUC* reporter. Transient expression assay showed that BIS3 was unable to transactivate the *BIS2* promoter in tobacco cells (Figure 6d). In addition, we measured the TIA pathway metabolites, such as loganic acid, secologanin, tabersonine, catharanthine and ajmalicine, in control and *BIS3*‐overexpressing flower petals. Accumulation of loganic acid increased significantly in *BIS3*‐overexpressing flowers, while the slight increase of secologanin, catharanthine, and tabersonine contents was not statistically significant compared to the control. Ajmalicine content was reduced in *BIS3*‐overexpressing flowers (Figure S4).

## 4 DISCUSSION

The advances in genome sequencing have significantly accelerated gene discovery and knowledge on genome organization, especially in non‐model plants. Unlike those of prokaryotes, genes in eukaryotic genomes are randomly distributed among the chromosomes. However, an increasing number of comparative genomic studies reveal that metabolic gene clusters are common in plants (Nutzmann et al., 2018). With a few exceptions (e.g., cytochrome P450 genes), a majority of the metabolic gene clusters comprise non‐homologous genes encoding enzymes for species‐specific metabolites. Genome sequence analysis has also identified TF gene clusters comprising of two or more homologous genes. Compared to the AP2/ERF clusters, clusters formed by other TFs, e.g., bHLH or MYB, are less characterized. Here, we discovered that the previously characterized *BIS1* and *BIS2* are joined by *BIS3* to form a subgroup‐IVa bHLH cluster (Figure 1). Comparative genomic analysis of primitive and higher plants revealed that subgroup‐IVa bHLH clusters appear to be present in the core angiosperms but not in lower plants (Figure 2). Genome‐wide analysis and evolutionary studies on bHLH gene families of different plant lineages suggest that expansion and diversity of bHLH genes have likely occurred after the split between green algae and land plant species, followed by a second expansion after the split between moss and vascular plants (Carretero‐Paulet et al., 2010; Feller et al., 2011). Therefore, bHLH clusters possibly have originated later in the evolution of higher plants through repeated gene duplication.

Transcriptomic resources have accelerated the identification of genes encoding enzymes or regulators in TIA and other metabolic pathways in plants. Candidate gene identification can often be achieved by coexpression analysis, based on the hypothesis that functionally related genes such as genes in a metabolic pathway, either in a cluster or randomly distributed in the genome, exhibit similar spatiotemporal expression profiles (Mutwil, 2020). In addition, previous studies suggest that transcription factors (TFs) regulating metabolic pathways often coexpress with genes encoding enzymes in the pathway (De Geyter et al., 2012). Supporting this notion, our coexpression analysis revealed that the enzyme‐encoding and regulatory genes in the iridoid branch of the TIA pathway are highly and preferentially expressed in roots (Figure 1). Biosynthesis of plant specialized metabolites, often acting as defense molecules under adverse conditions, is induced in response to many biotic and abiotic factors. The phytohormone JA and its methyl esters MeJA are key elicitors of diverse specialized metabolites, including SGAs, TIAs, terpenes, and nicotine (Cardenas et al., 2016; Lenka et al., 2015; Mertens, Pollier, et al., 2016; Patra et al., 2018; Paul et al., 2017; Shoji et al., 2010; Thagun et al., 2016). Here, we showed that similar to *BIS1* and *BIS2*, expression of *BIS3* was induced by MeJA (Figure 3). The JA‐response and similar spatiotemporal expression patterns as the iridoid pathway genes suggest that the *BIS* cluster coordinates the regulation of the TIA pathway.

The involvement of BIS3 in the regulation of the TIA pathway was further substantiated by the promoter activation assay in plant cells and transient overexpression in *C. roseus* flower petals. BIS3 significantly activates the promoters of *GES*, *G10H*, *8HGO*, *IS*, *7DLGT* and *7DLH*, key genes in the iridoid pathway. Transactivation activity of the *G10H* promoter by BIS1 (Figure S2) appears to be higher than that by BIS3 (Figure 4). This is possibly due to the sequence differences in the activation and DNA‐binding domains of the two TFs. Similar observations of differential activation of target gene promoters have been made for the members of the *ORCA* TF cluster in *C. roseus* (Paul et al., 2017) and *NIC2* ERFs in tobacco (Shoji et al., 2010), suggesting that individual TFs within a cluster may play different regulatory roles (Shoji & Yuan, 2021; Yuan, 2020). Similar to BIS1(Van Moerkercke et al., 2015), BIS3 does not activate the *IO* promoter in tobacco cells; however, overexpression of *BIS1* (Van Moerkercke et al., 2015), *BIS2* (Van Moerkercke et al., 2016) or *BIS3* (this study; Figure 6a) significantly upregulated *IO* expression in *C. roseus* hairy roots or flower petals. This is possibly due to 1) the presence of regulatory or enhancer elements further upstream in the *IO* promoter sequence and 2) the existence of a regulatory loop involving another factor that is activated by BIS TFs. BIS1 acts synergistically with BIS2 to activate some but not all iridoid pathway gene promoters in tobacco cells (Van Moerkercke et al., 2016). Here we demonstrated that *BIS3* overexpression induced the expression of *BIS1* and *BIS2*. Additionally, BIS3 acts synergistically with BIS1 or BIS2 to activate the *G10H* promoter in tobacco cells (Figure S2).

Mutation or deletion of the G‐box motif in the *G10H* promoter significantly reduced but not abolished its transactivation by BIS3 (Figure 5). Contraditive results have been reported for promoter activation by clade‐IV bHLH TFs in different plants, suggesting that the activation is most likely promoter‐context dependent (Mertens, Van Moerkercke, et al., 2016; Yamamura et al., 2015). For instance, mutation of the N‐box motif in the *C. roseus IS* promoter reduced but did not abolish its transactivation by BIS1 (Mertens, Van Moerkercke, et al., 2016). In rice, *CPS2* (*copalyl diphosphate synthase 2*) and *CYP99A2* (*cytochrome P450 monooxygenase 99A2*), involved in phytoalexin biosynthesis, are regulated by the clade‐IV bHLH TF DPF. Mutation of the N‐box motif in the *CPS2* promoter, but not the *CYPA99A2* promoter, completely eliminated the transactivation by DPF (Yamamura et al., 2015). In the *M. truncatula* saponin pathway, mutation of the N‐box motif in the *CYP93E2* promoter completely abolished its activation by the bHLH factor TSAR1 (Mertens, Pollier, et al., 2016). These findings suggest that G‐ or N‐box motifs are necessary but not exclusive for the activation of the *C. roseus G10H* and *IS* promoters by BISs and of the rice *CYP99A2* promoter by DPF. Other sequences in the promoters are likely involved in the regulation, as we demonstrated in the promoter deletion assay (Figure 5). The involvement of BIS3 in the regulation of the iridoid pathway was further demonstrated by overexpressing *BIS3* in *C. roseus* flower petals (Figure 6). *BIS3* overexpression significantly activated the expression of all seven iridoid pathway genes. The accumulation of loganic acid increased significantly in *BIS3*‐overexpressing flowers. Collectively, our observations authenticate the role of BIS3 in the regulation of the iridoid branch of the TIA pathway in *C. roseus*. Other TIA pathway metabolites, such as secologanin, tabersonine and catharanthine, did not show significant increase in *BIS3*‐overexpressing flowers (Figure S4). This is possibly due to the modest increase of expression of the indole pathway genes (*TDC*, *LAMT*, and *STR*) in *BIS3*‐overexpressing flowers compared to that of iridoid pathway genes (Figure 6). In addition, expression of *ORCAs* was either reduced or not significantly altered in *BIS3*‐overexpressing flowers (Figure S3). Similar to *BIS3*, *BIS1* overexpression in *C. roseus* flowers did not result in increased accumulation of tabersonine and catharanthine in flowers (Schweizer et al., 2018).

In conclusion, our work establishes the three‐member BIS TF cluster in *C. roseus*. Positive amplification and negative regulatory loops are evident in many metabolic and phytohormone signaling pathways. A regulatory loop between BIS3 and BIS1 or BIS2 seems to exist although likely to involve additional regulatory factors (Figure 6c,d). Although *BIS2* expression was significantly induced in *BIS3*‐overexpressing petals, BIS3 was unable to directly activate *BIS2* promoter in tobacco cells. This is similar to the previous study where BIS1 is unable to activate the *BIS2* promoter, although *BIS2* expression was significantly induced by BIS1, possibly due to 1) the presence of regulatory or enhancer elements further upstream in the promoter sequence and 2) the existence of a regulatory loop involving another factor that is activated by BIS TFs. The relationship among BIS1, BIS2, and BIS3 with regard to mutual regulation remains to be further explored. To date, only a few bHLH TF clusters have been functionally characterized. The characterization of the BIS cluster extended our understanding of bHLH TF clusters. Furthermore, the regulation of saponin biosynthesis in *M. truncatula* (Mertens, Pollier, et al., 2016; Ribeiro et al., 2020) and *C. quinoa* (Jarvis et al., 2017) as well as TIA biosynthesis in *C. roseus* (Van Moerkercke et al., 2015, 2016) (this study) by subgroup‐IVa bHLH TF clusters, suggests that an evolutionarily conserved regulatory mechanism modulates biosynthesis of specialized metabolites in plants.

## CONFLICT OF INTEREST

The authors declare no competing financial interests.

## AUTHOR CONTRIBUTIONS

L.Y., S.P., and S.K.S. designed the research; S.K.S., B.P., P.P., Y.L., and S.P. performed experiments; S.K.S., B.P., and S.P. analyzed data; and L.Y., S.K.S., and S.P. wrote the paper.

## Supporting information

Fig S1‐S4‐Table S1
