# Supplementary material for: BHLH IRIDOID SYNTHESIS 3 is a member of a bHLH gene cluster regulating terpenoid indole alkaloid biosynthesis in Catharanthus roseus
Source: Plant Direct. 2021 Jan 25;5(1):e00305. doi: 10.1002/pld3.305 (PMC7833464; doi:10.1002/pld3.305)
Supplement: Supplementary file 1 — Fig S1‐S4‐Table S1 [file PLD3-5-e00305-s001.pdf]

Supplementary information

BHLH IRIDOID SYNTHESIS 3 is a member of a bHLH gene cluster regulating terpenoid indole alkaloid biosynthesis in *Catharanthus roseus*

Sanjay Kumar Singh[2], Barunava Patra[2], Priyanka Paul[1], Yongliang Liu[2,3], Sitakanta Pattanaik[2], and Ling Yuan[1,2,3]

[1]Department of Plant and Soil Sciences, and [2]Kentucky Tobacco Research & Development Center, University of Kentucky, 1401 University Drive, Lexington, KY 40546 USA

[3]South China Botanical Garden, Chinese Academy of Sciences, Guangzhou, China

Authors for correspondence:

Sitakanta Pattanaik and Ling Yuan

Tel: 001 859 257 3586 / 859 257 4806

Email: spatt2@uky.edu; lyuan3@uky.edu;

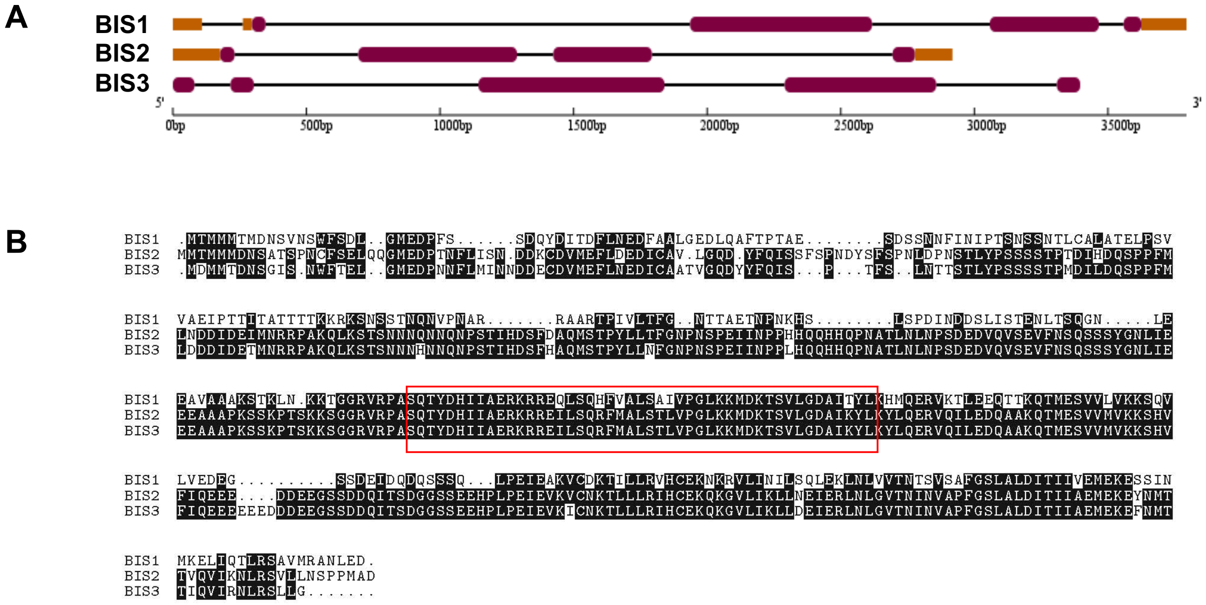

Fig. S1. Sequence analysis of BISs genes. **(A)** Gene structure of the BISs. The orange boxes indicate upstream/downstream region, brown color boxes for exons and straight lines represent introns. **(B)** Amino acid sequence alignment of the BISs. The alignment was performed with the CLUSTALO program using the default settings. The conserved amino acid residues are highlighted in black. The location of the conserved basic helix-loop-helix (bHLH) domains is indicated.

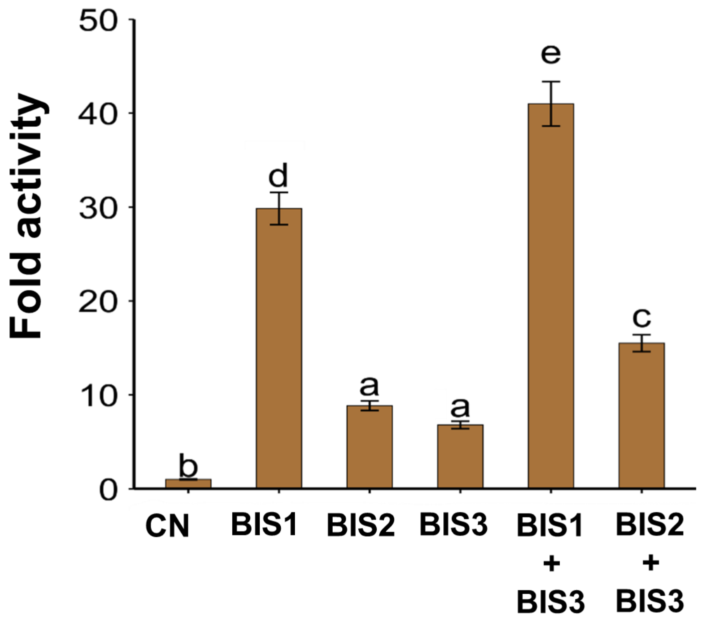

Figure S2 Transactivation of the *G10H* promoter by BIS1, BIS2 and BIS3 in tobacco cells. The *G10H* promoter fused to *luciferase* (*LUC*) reporter were electroporated into tobacco protoplasts either alone or with different effector plasmid (*BIS1/BIS2/BIS3*). The *CaMV*35S-*GUS* reporter served as an internal control. Luciferase activity was normalized against GUS activity. Control (CN) represents the reporter alone without effectors. Data represent the means±SD from three biological replicates. Different letters denote statistical differences as assessed by one-way ANOVA and Tukey HSD test, $p < 0.05$.

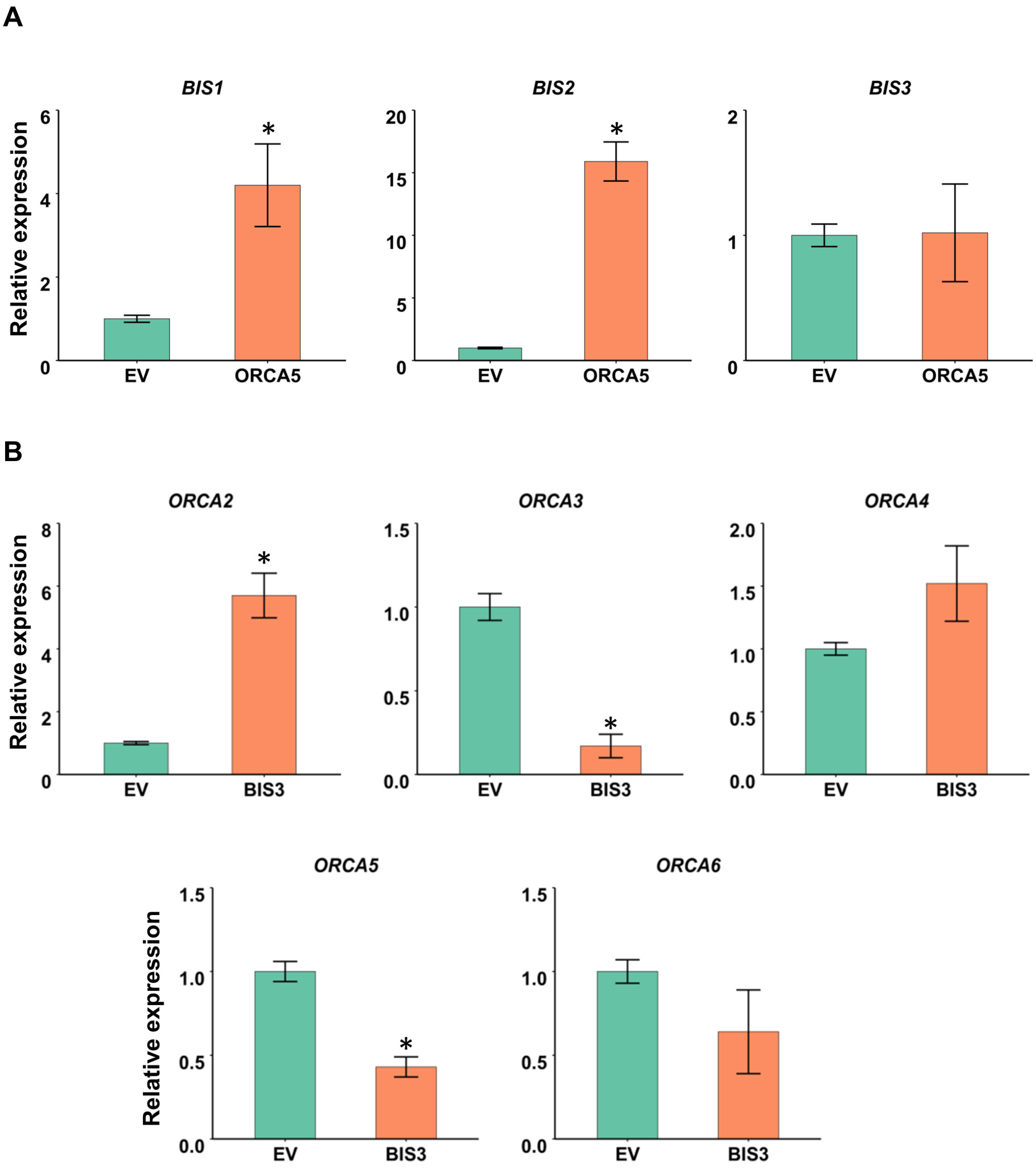

Fig. S3 Expression of *BIS* in *ORCA5*- and *ORCAs* in *BIS3*-overexpressing flower petals. Expression of *BIS1*, *BIS2* and *BIS3* in *ORCA5*-overexpressing (A) and *ORCAs* (*ORCA2*, *ORCA3*, *ORCA4*, *ORCA5* and *ORCA6*) in *BIS3*-overexpressing (B) flower petals relative to empty vector (EV) as measured by RT-qPCR. Data represent the means±SD from three biological replicates. Statistical significance was calculated using the Student's *t-test*: * $P < 0.05$

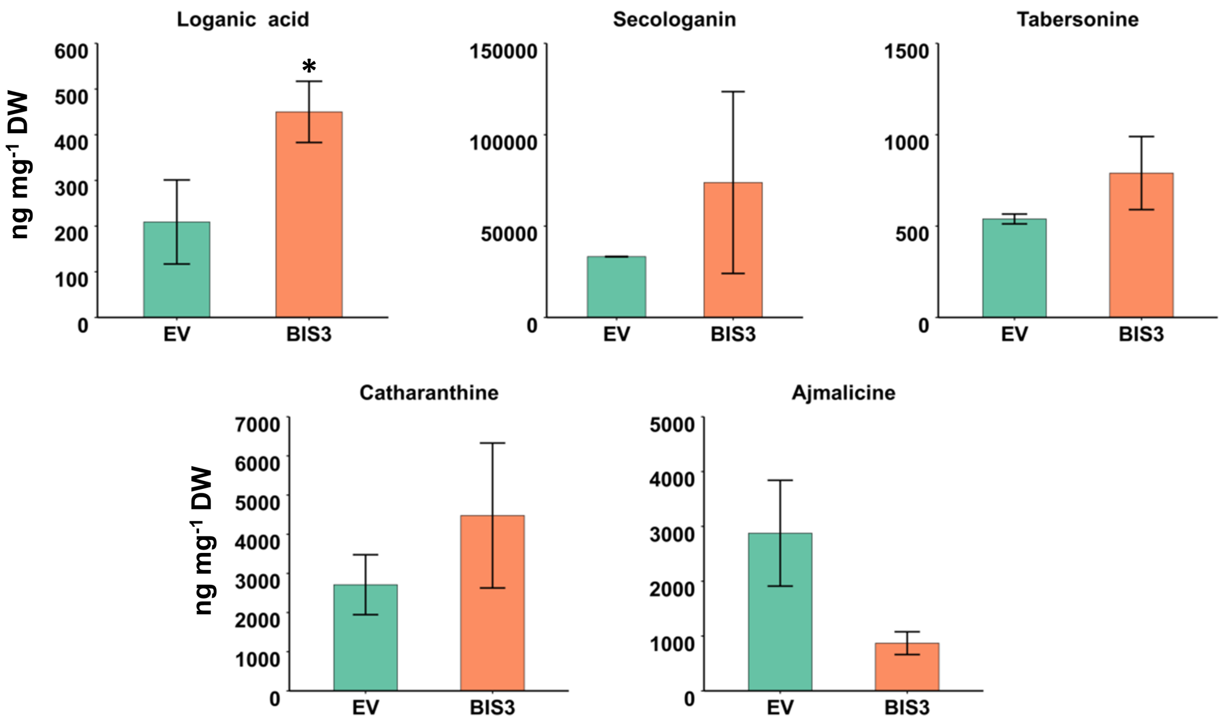

Fig. S4 Measurements of TIA pathway metabolites in empty vector (EV) and *BIS3*-overexpressing flower petals. Measurements of loganic acid, secologanin, tabersonine, catharanthine and ajmalicine in empty vector (EV) and *BIS3*-overexpressing flower petals. Alkaloids were extracted and analyzed by LC-MS/MS, and the concentrations of the alkaloids were estimated based on peak areas compared to standards. Alkaloid concentrations are indicated in ng mg_1 dry weight (DW). Data represent the means±SD from three biological replicates. Statistical significance was calculated using the Student's *t-test*: * $P < 0.05$

Supplemental Table 1 Primers used in this study

Gene name	Forward primer (5' - 3')	Reverse primer (5' - 3')
GES	ATCAAGTTCTTGCGCCAACC	TCATCTTCAAGGCCTCCAATGG
G10H	TTATTCGGATTCTGCCAAGG	TCCCCAAAGTGAATCGTCAT
8HGO	TGCTTTGTGCCAGTTTGTGC	ATGCGAGGGAACAATGGAAC
IS	CCACATGATTCGCCTTTTACCG	AAACCCGAAAACCAGAGCTG
IO	TGTGTGGGATTTCCTTTGGC	TTGTGGTTTAACGCCAGCAC
7DLGT	TCGCAAACACATGCCAAGAC	TGCATCAAGCCAATCCACAC
7DLH	TTTGCAGAGGGAGTTCTCAAGG	TGGCCAATGCACATTCTTGG
BIS1	ACCAGCGTTTCAGCTTTTGG	ATTTGCACGCATGACAGCTG
BIS2	ACGTCCCCTAATTGCTTCTC	GTCTTGACCAAGAACTGCAC
BIS3	AACTGGGAATGGAGGATCCT	TCTTGACCAACAGTTGCTGC
ORCA2	TGCGGGAGAACAAGAAGAAG	TTCGATCTCTGCTCACATCC
ORCA3	CGGGATCCGAAATACAGAAA	GCCCTTATACCGGTTCCAAT
ORCA4	ATAGTAGTACTGCCGCCGAAAG	ATCTCCGCCGCAAATTTTCC
ORCA5	TCTTTCAACGGAGGTTAACGG	AATGTTGTCTCCAGGGCTTG
ORCA6	TGGCTTGGGACTTACGAAAC	GCTCCACGCAACTTAAAAGC
TDC	ATCCGATCAAACCCATACCA	CGTCATCCTCGACCATTTTT
LAMT	AGCCAAGGCAGTGATTGTTG	CTGCAATGCGGAAAGGTTTG
SLS	GTTCCTTCTCACCGGAGTTG	CCCATTTGGTCAACATGTCA
STR	ACCATTGTGTGGGAGGACAT	ATTTGAATGGCACTCCTTGC
RPS9	GAGGGCCAAAACAAACTTGA	CCCTTATGTGCCTTTGCCTA
EF1œ	TACTGTCCCTGTTGGTCGTG	AAGAGCTTCGTGGTGCATCT
